# Cough reflex sensitivity improves with speech language pathology management of refractory chronic cough

**DOI:** 10.1186/1745-9974-6-5

**Published:** 2010-07-28

**Authors:** Nicole M Ryan, Anne E Vertigan, Sarah Bone, Peter G Gibson

**Affiliations:** 1 Centre for Asthma and Respiratory Diseases, School of Medicine and Public Health, The University of Newcastle, Newcastle, 2308, NSW, Australia; 2 Department of Respiratory and Sleep Medicine, Hunter Medical Research Institute, John Hunter Hospital, Newcastle, 2310, NSW, Australia; 3 Department of Speech Pathology, John Hunter Hospital, Newcastle, 2310, NSW, Australia

## Abstract

**Rationale:**

Speech language pathology is an effective management intervention for chronic cough that persists despite medical treatment. The mechanism behind the improvement has not been determined but may include active cough suppression, reduced cough sensitivity or increased cough threshold from reduced laryngeal irritation. Objective measures such as cough reflex sensitivity and cough frequency could be used to determine whether the treatment response was due to reduced underlying cough sensitivity or to more deliberate control exerted by individual patients. The number of treatments required to effect a response was also assessed.

**Objective:**

The aim of this study was to investigate subjective and objective measures of cough before, during and after speech language pathology treatment for refractory chronic cough and the mechanism underlying the improvement.

**Methods:**

Adults with chronic cough (n = 17) were assessed before, during and after speech language pathology intervention for refractory chronic cough. The primary outcome measures were capsaicin cough reflex sensitivity, automated cough frequency detection and cough-related quality of life.

**Results:**

Following treatment there was a significant improvement in cough related quality of life (Median (IQR) at baseline: 13.5 (6.3) vs. post treatment: 16.9 (4.9), p = 0.002), objective cough frequency (Mean ± SD at baseline: 72.5 ± 55.8 vs. post treatment: 25 ± 27.9 coughs/hr, p = 0.009), and cough reflex sensitivity (Mean ± SD log C5 at baseline: 0.88 ± 0.48 vs. post treatment: 1.65 ± 0.88, p < 0.0001).

**Conclusions:**

This is the first study to show that speech language pathology management is an effective intervention for refractory chronic cough and that the mechanism behind the improvement is due to reduced laryngeal irritation which results in decreased cough sensitivity, decreased urge to cough and an increased cough threshold. Speech language pathology may be a useful and sustained treatment for refractory chronic cough.

**Trial Registration:**

Australian New Zealand Clinical Trials Register, ACTRN12608000284369.

## Introduction

Chronic cough that persists despite medical treatment (termed refractory cough) is a difficult problem frequently associated with increased cough reflex sensitivity [1-3]. Management using speech language pathology is effective for both refractory cough and its associated voice disorder [4,5] but the mechanism behind the symptom improvement has yet to be determined. Cough reflex hypersensitivity plays an important role in chronic cough [6,7], and it was hypothesised that speech language pathology would either increase the threshold for cough or reduce cough sensitivity [4]. These effects could be achieved by either a behavioural approach to cough suppression or improved vocal hygiene leading to reduced laryngeal irritation.

This study sought to investigate capsaicin cough reflex sensitivity and automated cough frequency monitoring in patients with refractory chronic cough undergoing speech language pathology intervention. Cough reflex sensitivity testing and cough frequency monitoring are two objective measures allowing standardized assessment as well as providing an understanding of possible mechanisms of effect. Capsaicin is an extract of hot peppers and is commonly used as a tussive agent in clinical research because it induces cough in a safe, dose-dependent and reproducible manner [8-10]. Our aim was to objectively measure changes in cough reflex sensitivity and cough frequency prior to, during and after a speech language pathology treatment programme for refractory cough.

It was hypothesised that speech language pathology intervention for chronic cough would result in decreased cough reflex sensitivity, reduced cough frequency, improvement in clinical outcome and improvement in cough and laryngeal subjective measures. We also sought to determine how many treatment sessions a patient required to show an improvement and if these benefits were maintained post intervention.

## Methods

A previous pilot study compared 2 behavioral approaches (isolated cough suppression techniques and supportive counselling) for refractory chronic cough (CC) to a CC control group and showed that there was no change in cough reflex sensitivity (CRS) measured as C5 after 1 hour of intervention. These were used to establish the current study in the following ways;

1) C5 does not respond to isolated behavioural approaches,

2) C5 does not change after 1 × 1 hour session of an isolated behavioural approach, and, 3) CRS testing measured as C5 is a highly reproducible test.

### Participants

Adult non-smokers (n = 17) with chronic persistent cough that was refractory to medical assessment and treatment [11,12] and who were referred for speech language pathology management for cough [4] were eligible for the study. All participants provided written informed consent for this study, which was approved by the University of Newcastle's Human Research Ethics Committee and the Hunter New England Human Research Ethics Committee. **"For detailed description of the participants, procedures, and analysis, see additional file **1: **Participant details and results."**

### Study Design

Participants attended for a maximum of 6 visits (a baseline visit, up to 4 treatment visits and a post treatment visit) over a period of 14 to18 weeks. At visit 1, there was a voice assessment by a qualified speech language pathologist. This involved a clinical case history, symptom frequency and severity rating [13], auditory perceptual voice analysis and instrumental voice analysis utilizing acoustic and electroglottographic assessment. The auditory perceptual analysis was conducted utilizing the Perceptual Voice Profile by Oates and Russell [14] whereby 15 perceptual parameters of voice pitch, loudness and quality are rated on a severity scale from *normal *to *severe*. A clinical research officer then administered several questionnaires, [15-20] and conducted cough reflex sensitivity with capsaicin testing [8,21] and cough frequency by Leicester Cough Monitor [22] during the visit period. Visits 2-5 consisted of a 30 minute published speech language pathology programme for chronic persistent cough [4] followed by cough reflex testing and cough frequency. A post treatment visit was conducted 2 to 3 weeks after the final speech language pathology programme session (Visit 6) for objective cough monitoring.

### Speech Pathology treatment programme for chronic persistent cough

The speech pathology programme for chronic cough has been described previously [4] and consisted of four components: (a) education, (b) specific cough suppression strategies such as the Cough Suppression Swallow, Cough Control Breathing or paradoxical vocal fold movement release breathing techniques, (c) vocal hygiene training, and (d) psychoeducational counselling. All participants received each of the four components of the program.

### Capsaicin Cough Reflex Sensitivity (CRS) testing [8,21]

Capsaicin CRS was performed as previously reported with the addition of a participant urge-to-cough score [23] where the participant was asked to rate their urge to cough after each dose inhalation of capsaicin according to a modified Borg scale where 0 = "No urge to cough" up to 10 = "Maximum urge to cough".

### Leicester Cough Monitor (LCM) [22]

The LCM is a digital ambulatory cough monitor and external free-field microphone [22]. This was attached to the participant at the beginning of each objective cough measurement visit and removed at the end of the visit. The cough frequency collection period therefore encompassed a recording time of about one hour in which questionnaires and cough reflex testing were performed. This measurement was used to complement the cough reflex sensitivity test by measuring any change in the patient's frequency of coughing after speech pathology intervention. Data stored on the recorder was downloaded onto a computer where it was analysed by an automated cough detection algorithm (the Leicester Cough Algorithm, [24,25]). Cough was defined as a characteristic explosive sound (throat clears were classified by operator input as a "non-cough" to be consistent with CRS cough counting) and reported as coughs/hour.

### Analysis

All analyses were performed using statistical and data analysis software STATA (Statacorp, Texas, USA). Comparisons of log cough sensitivity (measured as C5 and cough threshold) between baseline and each visit was undertaken using a generalised linear mixed model (GLMM) with a random intercept term which takes into account the repeated observations on individuals. Standard errors were estimated using bootstrapping [26] and results were expressed as Mean ± SD. Parametric bootstrap is a practical tool for addressing problems associated with inference from GLMMs by producing sensible estimates for standard errors. Similar models were used to examine the change in cough frequency although data was assumed to have a Poisson distribution. P values < 0.05 were considered significant.

Figures were produced using GraphPad Prism 4 (GraphPad Software, Inc, California, USA).

## Results

Seventeen participants (8 male and 9 female) with a chronic persistent cough participated in the study. The participants had a median (IQR) cough duration of 60 (147) months and age of 61 (20) years with normal spirometry [Table 1]. Co-morbidities included gastroesophageal reflux disease (n = 10), asthma (n = 2), eosinophilic bronchitis (n = 1) and rhinitis (n = 8). Treatment trials were implemented for these conditions including proton pump inhibitors for gastroesophageal reflux disease, inhaled corticosteroids for asthma and eosinophilic bronchitis, and nasal corticosteroid and/or antihistamine for rhinitis. When cough proved refractory to these treatments, speech language pathology was implemented. An initial participant cough assessment performed by a speech language pathologist found that 63% of participants had abnormal auditory perceptual voice analysis. There was also a high incidence of abnormal acoustic and electrographic instrumental voice analysis [Table 1]. The number of treatment sessions for each participant was determined by their response to the therapy; specifically this included the effectiveness of the technique, the participant's ability to perform and implement the technique appropriately, their understanding of the rationale for the treatment, and availability to attend treatment sessions. Generally, participants attended 3 (n = 4) or 4 (n = 9) speech treatment sessions while 3 participants responded rapidly and only required 2 treatment sessions. One participant only received 1 treatment session due to personal reasons. Participant compliance was evaluated through informal interview between the participant and speech pathologist at the beginning of each session. Participant compliance with the speech language pathology programme was determined to be "good" in 53% of the participants; "partial" in 35% and 12% were classified as non-adherent.

**Table 1:** Subject Characteristics.

Subject Characteristics		Normal Range
Number, (M/F)	17 (8/9)	

Age, years	61 (20)	

Age Range, years	34-83	

Cough Duration, months	60 (147)	

FEV1, %predicted	88.2 (16.7)	

FVC, %predicted	88.5 (20.3)	

Auditory perceptual voice analysis, % abnormal	63	

Maximum phonation time, seconds	12.8 (8.9)	>15
Range, seconds	1 - 26	

Jitter, percent	1.7 (1.6)	< 1
Range, percent	0.4 - 6.5	

Harmonic to noise ratio, dB SPL	15.9 (3.8)	> 20
Range, dB SPL	10 - 24.7	

Speaking fundamental frequency, Hertz	Female: 178 (20)	180 - 200 (female)
Range, Hertz	154 - 198	90-130 (male)
Range, Hertz	Male: 110 (14)97 - 133	

Closed phase, percent	43.5 (6.4)	44.5
Range, percent	32 - 53	

Median (IQR) unless otherwise stated.

**Figure 1:**
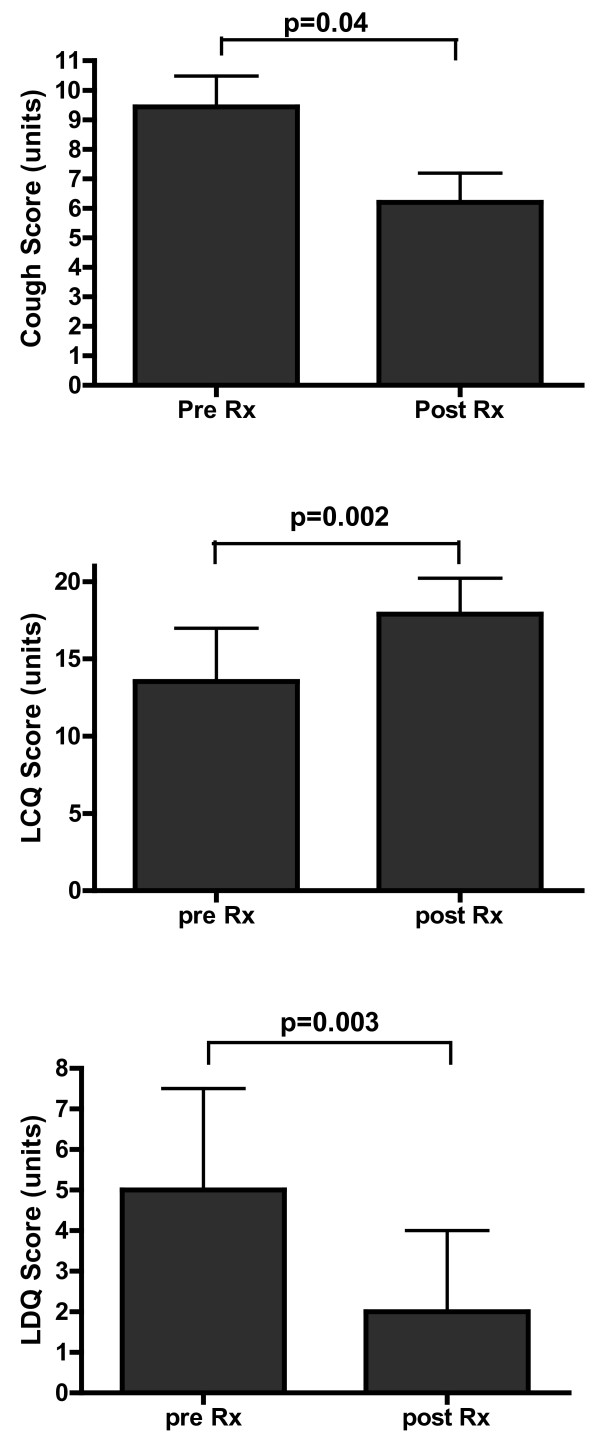
**Cough subjective measures of a) Cough Score b) Cough Quality of Life and c) Laryngeal Dysfunction (Baseline vs Post Treatment).** Effect of speech-language pathology treatment on refractory chronic cough outcomes of a) Cough symptoms scores (Mean ± SD). b) Leicester cough questionnaire Median (IQR) and c) Laryngeal dysfunction questionnaire Median (IQR).

**Figure 2:**
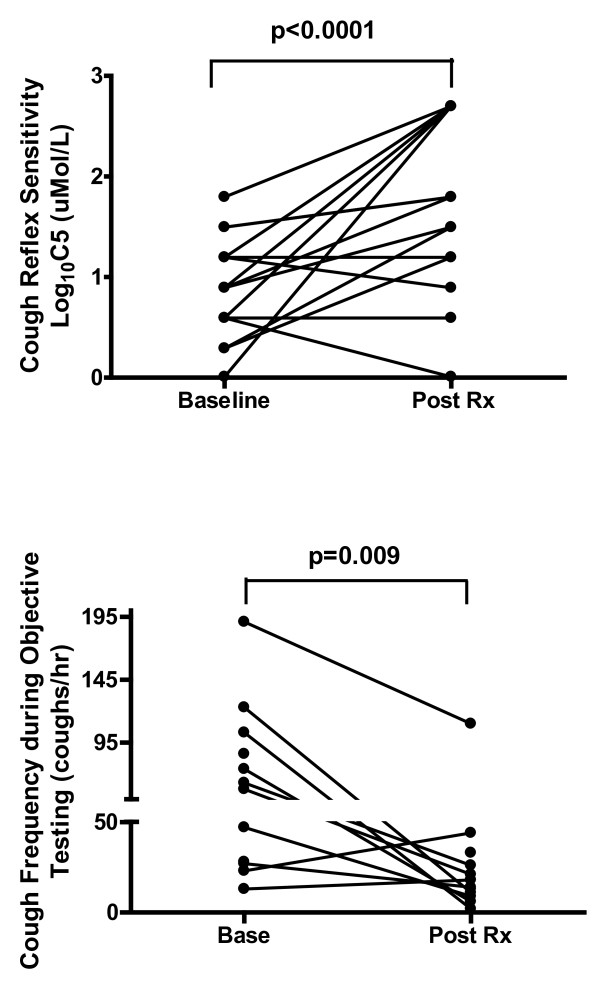
**Objective cough measures of a) Cough Reflex Sensitivity (C5) and b) Cough Frequency (Baseline vs Post Treatment).** Effect of speech-language pathology treatment on refractory chronic cough outcomes of a) Log Cough Reflex Sensitivity at baseline (Base), and post treatment (Post Rx) for individual data. C5 = capsaicin dose to elicit 5 or more coughs 30 sec after dose administered. b) Cough Frequency at baseline (Base), and post treatment (Post Rx).

Participants responded to the treatment with a significant improvement in cough-related quality of life (LCQ, p = 0.002), laryngeal dysfunction symptom questionnaire score (LDQ, p = 0.003), cough score, p= 0.04 and total symptoms score, p = 0.002 [Table 2, Figure 1]. There was a significant improvement in cough reflex sensitivity measured as C5 with speech language pathology treatment for chronic persistent cough. Cough reflex sensitivity was heightened at baseline, Mean ± SD log C5 0.88 ± 0.48 and significantly improved with treatment to log C5 1.65 ± 0.88, p < 0.0001 [Individual log C5 data (baseline v post treatment) represented in Figure 2a]. Improvements in cough reflex sensitivity were apparent after each visit: treatment visit 1, Mean ± SD log C5 (T1) 1.18 ± 0.62, p = 0.023, treatment visit 2 (T2) log C5 (T2) 1.46 ± 0.78, p < 0.0001, treatment visit 3 (T3) log C5 1.45 ± 0.68 p < 0.0001, and treatment visit 4 (T4) log C5 1.53 ± 0.93, p < 0.0001 [Table 3]. These results indicate that the improvement in cough reflex sensitivity occurred after the first treatment visit, increased at subsequent treatment visits (significant treatment response attained after 2 treatments and maximum treatment response after 4 treatments) and that the effect was sustained at the post treatment visit.

**Table 2:** Questionnaire Scores.

Measurement	Baseline	Post Treatment	p
Cough Symptom Score (Mean ± SD)	9.4 ± 4.2	6.2 ± 3.8	0.04

Total Symptom Score	30 (23.5)	16 (10)	0.002

LCQ Score	13.5 (6.3)	16.9 (4.9)	0.002

GORD Score	14.5 (6.0)	15.5 (11.0)	0.96

Snot-20 Score	1.3 (1.5)	0.6 (1.3)	0.11

LDQ Score	5 (4)	2 (2)	0.003

HADS Anxiety Score	9.5 (2.0)	11.0 (4.5)	0.33

HADS Depression Score	10 (2)	10 (6)	0.34

Median (IQR) unless otherwise stated.

LCQ = Leicester Cough Questionnaire

GORD = Gastroesophageal reflux disease

Snot-20 = 20-item Sino-Nasal Outcome Test

LDQ = Laryngeal Dysfunction Questionnaire

HADS = Hospital Anxiety and Depression Scale

**Table 3:** Capsaicin Cough Reflex Sensitivity Test, Urge-to-Cough and Leicester Cough Monitor Testing.

Measurement	Baseline	T1	T2	T3	T4	Post Treatment	P*
Log CRS, C5 μMol/L	0.88 ± 0.48	1.18 ± 0.62	1.46 ± 0.78	1.45 ± 0.68	1.53 ± 0.93	1.65 ± 0.88	< 0.0001

Cough Frequency (coughs/hr)	72.5 ± 55.8	42.5 ± 60.5	63.0 ± 78.9	48.7 ± 36.8	29.4 ± 18.4	25.0 ± 27.9	0.009

Log Cough Threshold μMol/L	0.47 ± 0.38	0.72 ± 0.60	0.80 ± 0.60	0.69 ± 0.23	0.66 ± 0.65	1.14 ± 0.76	0.001

Urge to Cough Score, Median (IQR)	5 (1)	3.5 (4.0)	3 (5)	1.5 (3.0)	0.5 (1.0)	1 (4)	0.01

Mean ± SD unless otherwise indicated.

* Baseline vs Post Treatment T = Treatment Post = Post Treatment

There was also a significant decrease in cough frequency with the speech language pathology treatment for chronic persistent cough. The cough count at baseline was reduced after treatment: Mean ± SD cough frequency, 72.5 ± 55.8 vs. 25 ± 27.9 coughs/hr, p = 0.009 [Individual cough frequency data (baseline v post treatment) represented in Figure 2b] and the cough count tended to reduce each treatment visit and reached significance after treatment visit 3: cough frequency Mean ± SD treatment visit 1 (T1) 42.5 ± 60.5 coughs/hr, p = 0.23, treatment visit 2 (T2) 63.0 ± 78.8 coughs/hr, p = 0.34, treatment visit 3 (T3) 48.7 ± 36.8 coughs/hr, p = 0.005 and treatment visit 4 (T4) 29.4 ± 18.4 coughs/hr, p < 0.0001 [Table 3]. The effect of the treatment programme on cough frequency was not as immediate as the effect on C5 with a significant result occurring after treatment visit 3 rather than at visit 1. The effect of treatment on cough frequency continued for treatment visit 4 (maximum treatment response) and was sustained at the post treatment visit.

**Figure 3:**
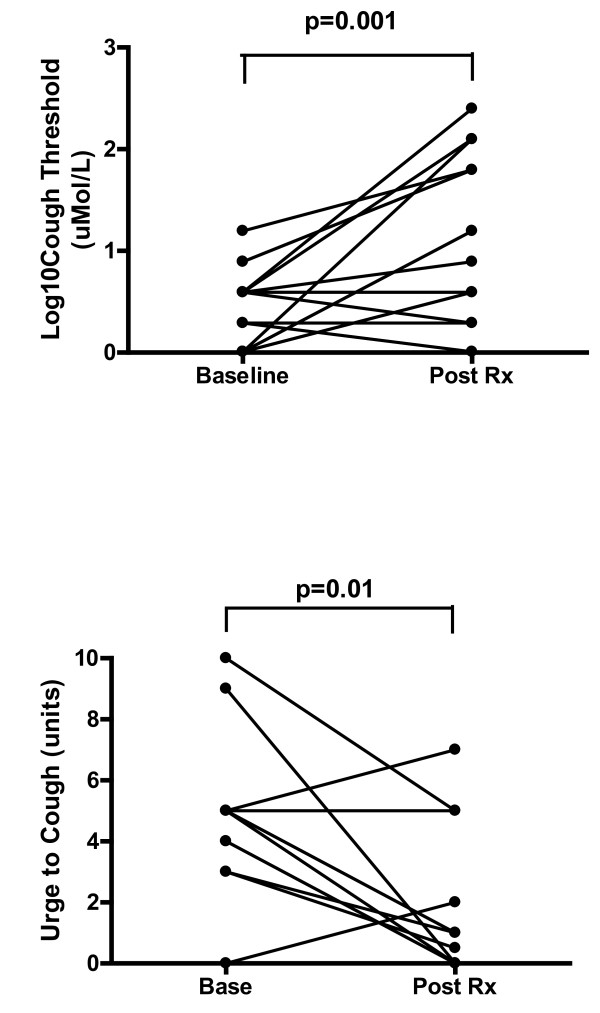
**Objective measure-Cough Threshold (a) and Participants urge-to-cough at C5 (b) (Baseline vs Post Treatment).** Effect of speech-language pathology treatment on refractory chronic cough outcomes of a) Log Cough Threshold at baseline (Base), and post treatment (Post Rx). b) Urge to Cough score at baseline (Base), and post treatment (Post Rx).

Cough threshold at baseline was Mean ± SD log CT 0.47 ± 0.38 and was significantly altered during treatment: treatment visit 1, cough threshold (T1) log CT 0.72 ± 0.60, p = 0.024, treatment visit 2 (T2) log CT 0.80 ± 0.60, p = 0.025, treatment visit 3 (T3) log CT 0.69 ± 0.23, p = 0.002, until maximum effect had been achieved with no significant change at treatment visit 4 (T4) log CT 0.66 ± 0.65, p = 0.122. After completion of therapy, cough threshold improved significantly: log CT 1.14 ± 0.76, p = 0.001 [Individual cough threshold data (baseline v post treatment) represented in Figure 3a].

There was a significant decrease in urge-to-cough with the speech language pathology treatment for chronic persistent cough. The urge-to-cough at baseline was reduced after treatment: Median (IQR), 5 (1) vs. 1 (4), p = 0.01 [Individual urge to cough data (baseline v post treatment) represented in Figure 3b] and the urge-to-cough tended to reduce after each treatment visit and reached significance after treatment visit 3: urge to cough Median (IQR) treatment visit 1 (T1) 3.5 (4), p = 0.38, treatment visit 2 (T2) 3 (5), p = 0.61, treatment visit 3 (T3) 1.5 (3), p = 0.005 and treatment visit 4 (T4) 0.5 (1), p = 0.24.

## Discussion

This is the first study to objectively assess response to a speech language pathology programme for refractory chronic cough using measures of cough sensitivity and cough frequency. We have shown that patients with refractory chronic cough have significantly decreased cough sensitivity and cough frequency together with an improvement in clinical outcome and cough and laryngeal symptoms following the speech language pathology intervention. Participants had an early symptom response to the speech language pathology program that was further improved upon throughout subsequent treatment sessions. Generally, a patient needed 3 to 4 treatment sessions and the response was maintained after the intervention ceased.

The speech language pathology program for refractory CC includes several components, and from a previously conducted pilot study *(presented in the additional data file 1: Participant details and results) *it was found that isolated components such as specific cough suppression techniques or counselling were not enough for a patient to achieve a clinical response. In a previous study [27], we showed the benefit of the speech language pathology program combined with a cough diagnostic and treatment algorithm [12] on cough reflex sensitivity in chronic persistent cough patients with paradoxical vocal fold movement (PVFM). This study expands on those results by treating patients with cough that is refractory to usual medical care with or without the presence of PVFM and investigating the mechanism of action. Our aim was to objectively measure changes in cough reflex sensitivity and cough frequency prior to speech language pathology program, during the speech pathology language program and at a post-treatment visit. We found that both cough frequency and cough sensitivity improved progressively with the speech language pathology program. Statistically significant improvements in cough reflex sensitivity were apparent after 1 treatment session, and this resulted in significant reduction in cough frequency after 3 sessions.

Within the large population of patients with CC, there is a small subgroup that does not respond to usual medical treatment [3,28]. In the past this group has been referred to as chronic idiopathic cough [1]. This group has been shown to have increased sensitivity to capsaicin challenge indicating a heightened cough reflex. The typical refractory cough patient will have coughing bouts triggered by normal daily activities such as exposure to aerosols, perfumes, cold air or when talking or laughing. Patients also describe a 'tickle, irritation, lump or blockage' in the throat preceding the urge to cough. While the mechanism/s of chronic idiopathic cough are currently unknown it has been proposed that chronic idiopathic cough maybe similar to other sensory hyperalgesias, where there is a long-standing reduction in sensory nerve threshold to stimulation [29,30]. We previously showed that up to 60% of refractory or idiopathic cough can be associated with paradoxical vocal fold movement - a sensory laryngeal hypersensitivity with heightened cough reflex sensitivity and extrathoracic airway hyperresponsiveness [7]. Both extrathoracic airway hyperresponsiveness and cough reflex sensitivity respond to diagnostic medical treatment with the addition of speech language pathology in chronic cough, and in the current study we now extend that data to show that refractory cough with or without PVFM responds to speech language pathology program for cough that persists after usual treatments have been exhausted. This study investigated the mechanism of the improvement in sensory hyperresponsiveness in chronic idiopathic cough following a speech language pathology programme. The mechanism of the effect is due to a reduction in cough reflex sensitivity. The speech language pathology program has several components that include cough suppression behaviour and vocal hygiene training. Voluntary cough suppression does not appear to be the primary mechanism of effect since we saw the effect of the speech language pathology program on cough threshold during the treatment programme. This is also supported by a pilot study where we examined the individual speech language pathology program components and found no effect of the cough suppression component on cough reflex sensitivity.

The study does suggest that the effective speech language pathology programme components reduce cough reflex sensitivity. This effect could occur by improvement in vocal hygiene leading to reduce sensory nerve stimulation, and is supported by the improvements in C5 and urge to cough during the programme. It is also possible that the reduction in cough frequency subsequently reduces cough-related airway trauma, and this explains the delayed improvement in cough threshold.

In this study we used an open design with objective measures to assess outcome. Although a nonrandomized design is a limitation, our primary purpose was to treat refractory cough patients and determine their response to a therapy outside normal chronic cough treatment. We achieved this aim by using objective measures and presenting novel data showing that cough frequency and cough reflex hypersensitivity significantly improve after speech language pathology treatment. It is possible that a placebo effect such as cough suppression [31-33] may have influenced some of the measures used in this study. We believe however that this is unlikely as the majority of the subjects studied had a cough for more than 5 years duration and underwent numerous cough treatments prior to speech language pathology intervention. Also, if there was a placebo effect at work then an improvement in C5 and cough threshold may be seen but there would be no change in the subjects urge to cough [23,34] as seen here.

We did not find a heightened cough reflex sensitivity in CC females compared to CC males (power 90%) and this was consistent with our previous research [7,27] (for further results on this refer to additional file 1: Participant details and results). A gender difference in cough reflex sensitivity has been reported in some healthy subjects without cough [35,36] but not all [37] studies. We studied subjects representative of those with refractory chronic persistent cough. They were primarily middle-aged with a significant cough duration, had been treated for the usual causes of cough [12] and had not responded to those treatments. We assessed cough reflex sensitivity to capsaicin and cough frequency using validated techniques [8,21,22] and present novel data on how this group respond to speech language pathology treatment for chronic cough.

## Conclusion

In conclusion, this is the first study to show that speech language pathology management is an effective intervention for refractory chronic cough and that the mechanism behind the improvement is due to reduced laryngeal irritation which results in decreased cough sensitivity, decreased urge to cough and an increased cough threshold. This is accompanied with an improvement in cough symptoms, associated laryngeal symptoms, and cough quality of life. Speech language pathology may be a useful therapy for refractory chronic cough.

## List of Abbreviations

CC: Chronic Cough; CRS: Cough Reflex Sensitivity; C5: concentration of capsaicin required to elicit 5 or more coughs within 30 secs after dose administration; LCM: Leicester Cough Monitor; LCQ: Leicester Cough Questionnaire; GLMM: Generalized linear mixed model; LDQ: Laryngeal Dysfunction Questionnaire; PVFM: Paradoxical Vocal Fold Movement; IQR: InterQuartile Range; T1-T4: Treatment No.; GORD: Gastro Oesophageal Reflux Disease; Snot-20: 20-item sino-nasal outcome test; HADS: Hospital Anxiety and Depression Scale.

## Competing interests

The authors declare that they have no competing interests.

## Authors' contributions

NR, AV and PG planned the study. AV, SB recruited the subjects, NR performed the objective cough tests. NR, AV and SB performed questionnaires, collected data and calculated scores. NR analysed the data. AV, SB performed speech pathology treatment. AV participated in the manuscript drafting. PG participated in the data interpretation, manuscript drafting and coordination of the manuscript. All authors read and approved the final manuscript.

## Supplementary Material

Additional file 1 **Participant details, supplemental methods and results**. Click here for file
