# Supplementary material for: Cough reflex sensitivity improves with speech language pathology management of refractory chronic cough
Source: Cough. 2010 Jul 28;6:5. doi: 10.1186/1745-9974-6-5 (PMC2921346; doi:10.1186/1745-9974-6-5)
Supplement: Additional file 1 — Participant details, supplemental methods and results. [file 1745-9974-6-5-S1.DOC]

**Additional data file 1: Participant details, supplemental methods and results**

*Participants*

Adults (n=17) with chronic persistent cough that was refractory to medical assessment and treatment (1, 2) and who were referred for speech language pathology management for cough (3) were eligible for the study. They were aged between 18 and 80 years, non-smokers or ex-smokers with less than ten pack years, had no other active respiratory or cardiac disease, and had a normal chest radiograph.

*Study Design*

Participants attended for a maximum of 6 visits (a baseline visit, up to 4 treatment visits and a post treatment visit) over a period of 14 to18 weeks. At visit 1, there was a voice assessment by a qualified speech language pathologist. This involved a clinical case history, symptom frequency and severity rating (4), auditory perceptual voice analysis and instrumental voice analysis utilizing acoustic and electroglottographic assessment. The auditory perceptual analysis was conducted utilizing the Perceptual Voice Profile by Oates and Russell (5) whereby 15 perceptual parameters of voice pitch, loudness and quality are rated on a severity scale from *normal* to *severe*. A qualified clinical research officer then collected participant clinical history, current cough symptoms and medication use. A cough specific quality of life questionnaire (Leicester Cough Questionnaire, (LCQ)) (6), a gastroesophageal reflux disease (GORD) questionnaire (7), a rhinosinusitis questionnaire (Snot-20) (8), Asthma Control questionnaire (ACQ) (9), the Hospital Anxiety and Depression Scale (10) and a laryngeal dysfunction questionnaire (LDQ) (11) were then administered.

A baseline cough reflex sensitivity to capsaicin (CRS) (12, 13) and Urge-to-Cough score (14) were administered while the participant wore an automated cough monitor and microphone to record cough frequency (15) during the visit.

Visits 2-5 consisted of a published speech pathology programme for chronic persistent cough (3). Each treatment session lasted for approximately 30 minutes and was followed by cough reflex sensitivity testing with urge-to-cough score and objective cough monitoring of cough frequency.

A post treatment visit (visit 6) was conducted 2 to 3 weeks after the final treatment session with the speech language pathologist to ascertain whether the result was sustained once the one-on-one intervention had stopped. At this visit the participant repeated the same questionnaires as that for visit 1 followed by the capsaicin cough reflex sensitivity test, Urge-to-Cough score, and objective monitoring of cough frequency.

The education component was designed to explain that in contrast to acute cough, there is no physiological benefit from cough and that there are negative side effects to cough. The cough suppression strategies trained participants to identify precipitating warning signs for cough and then to substitute competing responses. These responses included the cough suppression swallow, relaxed throat breathing with abdominal support, paradoxical vocal fold movement release breathing or cough control breathing. Vocal hygiene training was designed to reduce laryngeal irritation and maintain adequate hydration. These strategies involved systemic and surface hydration and minimization of irritants such as alcohol, caffeine and laryngopharyngeal reflux. The final component was psycho-educational counselling which aimed to help participants to internalize control over the cough and view it as a behaviour occurring in response to laryngeal irritation rather than an external or chance event. It also involved setting realistic treatment goals, reframing their response to laryngeal irritation, and accepting the need to adhere to treatment and the requirement for daily practice.

*Speech Pathology treatment programme for chronic persistent cough*

The speech pathology programme for chronic cough has been described previously (3) and consisted of four components: (a) education, (b) specific cough suppression strategies, (c) vocal hygiene training, and (d) psychoeducational counselling. All participants received each of the four components of the program however the duration and frequency of each component was tailored to the individual patient. The education component was designed to explain that in contrast to acute cough, there is no physiological benefit from cough and that there are negative side effects to cough. The cough suppression strategies trained participants to identify precipitating warning signs for cough and then to substitute competing responses. These responses included the cough suppression swallow, relaxed throat breathing with abdominal support, paradoxical vocal fold movement release breathing or cough control breathing. Vocal hygiene training was designed to reduce laryngeal irritation and maintain adequate hydration. These strategies involved systemic and surface hydration and minimization of irritants such as alcohol, caffeine and laryngopharyngeal reflux. The final component was vocal hygiene training which aimed to help participants to internalize control over the cough and view it as a behaviour occurring in response to laryngeal irritation rather than an external or chance event. It also involved setting realistic treatment goals, reframing their response to laryngeal irritation, and accepting the need to adhere to treatment and the requirement for daily practice.

*Clinical Methods*

*Capsaicin Cough Reflex Sensitivity (CRS) testing* (12, 13)

Solutions of capsaicin (Sigma-Aldrich Co., Castle Hill, Australia) concentrations ranging from 0.98 to 500 µM were prepared daily. Subjects inhaled single breaths (from Functional Residual Capacity (FRC) to total lung capacity (TLC)) of capsaicin aerosol from a compressed air-driven nebulizer (model 646, Technipro, North Parramatta, Australia) controlled by a dosimeter (KoKo Digidoser 323200; Technipro Marketing Pty Ltd., Sydney, New South Wales, Australia). The inspiratory flow was standardized at 0.5L/s with an inspiratory flow regulator valve. Cough counting was done for 30s after exposure to each dose, and the investigation ended when the subject coughed five or more times in response to one dose, or received a dose of the highest concentration. Urge to cough score (14) was recorded at baseline, (after spirometry just before the first administration of the capsaicin solution challenge), again after the administration of each capsaicin solution and at the conclusion of the CRS test. The participant was asked to rate their urge to cough from a modified Borg scale with 0 being “no need to cough” upto 10 being “maximum urge to cough”.

*Leicester Cough Monitor (LCM)* (15)

The LCM is a digital ambulatory cough monitor that records sound from an external free-field microphone (Sennheiser MKE 2-ew Gold; Germany) positioned against the participant’s chest wall onto a digital sound recorder (dimensions 26.7x87x32 mm; iRiver iFP-799; iRiver America) at a sampling frequency of 16 kHz and with an encoding bit rate of 64 kbit.s^-1^ (15). This was attached to the participant at the beginning of each visit and removed at the end of the visit. Data stored on the recorder was downloaded onto a computer where it was analysed by an automated cough detection algorithm (the Leicester Cough Algorithm, (16, 17)). Cough was defined as a characteristic explosive sound (throat clears were classified by operator input as a “non-cough” to be consistent with CRS cough counting) and reported as coughs/hour. The algorithm identifies coughs as single events whether they occur as isolated events or in a cluster and reports the cough frequency as cough events and coughs/hour. This study reports cough frequency as coughs/hr.

**Results:**

We did not find a heightened CRS in CC females compared to CC males (power 90%, Figure 1S).

There was no significant effect of gender on cough reflex sensitivity before and after treatment [Table 1S].

Figure 1S. Pilot Study Results, CS = isolated cough suppression technique,

C = supportive Counselling, NI = no intervention. C5 = capsaicin dose to elicit 5 or more coughs 30sec after dose administered.

**References:**

1. Irwin RS, Madison JM. The diagnosis and treatment of cough. *N Engl J Med* 2000;343:1715-1721.

2. Kastelik JA, Aziz I, Ojoo JC, Thompson RH, Redington AE, Morice AH. Investigation and management of chronic cough using a probability-based algorithm. *Eur Respir J* 2005 25:235-243.

3. Vertigan AE, Theodoros DG, Gibson PG, Winkworth AL. Efficacy of speech pathology management for chronic cough: a randomised placebo controlled trial of treatment efficacy. *Thorax* 2006;61:1065-1069.

4. Vertigan A, Theodoros D, Gibson P, Winkworth A. Voice and upper airway symptoms in people with chronic cough and paradoxical vocal fold movement. *J Voice* 2007;21:361-383.

5. Oates J, Russell A. Learning voice analysis using an interactive multi-media package: Development and preliminary evaluation. *J Voice* 1998;12:500-512.

6. Birring SS, Prudon B, Carr AJ, Singh SJ, Morgan MDL, Pavord ID. Development of a symptom specific health status measure for patients with chronic cough: Leicester Cough Questionnaire. *Thorax* 2003;58:339-343.

7. Locke GR, Talley NJ, Weaver AL, Zinsmeister AR. A New Questionnaire for Gastroesophageal Reflux Disease. *Mayo Clinical Procedures* 1994;69:539-547.

8. Piccirillo JF, Merritt MG, Richards ML. Psychometric and clinimetric validity of the 20-item sino-nasal outcome test (Snot-20). *Otolaryngol Head Neck Surg* 2002;126:41-47.

9. Juniper EF, O'Byrne PM, Ferrie PJ, King DR, Roberts JN. Measuring asthma control. Clinic questionnaire or daily diary? *Am J Respir Crit Care Med* 2000;162:1330-1334.

10. Zigmond AS, Snaith RP. The hospital anxiety and depression scale. *Acta Psychiatr Scand* 1983;67:361-370.

11. Powell GH, Ryan NM, Taramarcaz P, Gibson PG. Development and validation of a vocal cord dysfunction questionnaire [abstract]. *Respirology* 2007;12 (Suppl. 1):A39.

12. Birring SS, Matos S, Patel RB, Prudon B, Evans DH, Pavord ID. Cough frequency, cough sensitivity and health status in patients with chronic cough. *Respir Med* 2006;100:1105-1109.

13. Dicpinigaitis PV. Short- and long-term reproducibility of capsaicin cough challenge testing. *Pulm Pharmacol & Therapeutics* 2003;16:61-65.

14. Davenport PW. Urge-to-cough: what can it teach us about cough? *Lung* 2008;186:S107-111.

15. Birring SS, Fleming T, Matos S, Raj AA, Evans DH, Pavord ID. The Leicester Cough Monitor: preliminary validation of an automated cough detection system in chronic cough. *Eur Respir J* 2008;31:1013-1018.

16. Matos S, Birring SS, Pavord ID, Evans DH. Detection of cough signals in continuous audio recordings using hidden Markov models. *IEEE Trans Biomed Eng* 2006;53:1078-1083.

17. Matos S, Birring SS, Pavord ID, Evans DH. An automated system for 24-hour monitoring of cough frequency: the Leicester cough monitor. *IEEE Trans Biomed Eng* 2007;54:1472-1479.

Table 1S. Cough reflex sensitivity, C5 for each visit according to Gender.

Mean ± SEM represented.

| **CRS, C5 (uMol/L)** | **Male** | **Female** | **p** |
| --- | --- | --- | --- |
| Baseline Visit | 14.6±20.2 | 12.0±11.7 | 0.75 |
| Treatment Visit 1 | 33.4±25.6 | 24.3±24.6 | 0.46 |
| Treatment Visit 2 | 118.5±189.9 | 107.8±168.9 | 0.91 |
| Treatment Visit 3 | 36.1±25.7 | 116.4±177.9 | 0.34 |
| Treatment Visit 4 | 99.3±131.5 | 175.3±251.7 | 0.65 |
| Post Treatment Visit | 222.7±259.5 | 137.8±206.8 | 0.48 |
